# Optimization design of sand-containing fog seal overlay on chip seal based on response surface methodology

**DOI:** 10.1371/journal.pone.0355403

**Published:** 2026-08-12

**Authors:** Jun’an Lei, Xiaoyu Jiang, Qiliang Liu, Yongtai He

**Affiliations:** 1 School of Civil Engineering and Architecture, HuBei University of Arts and Science, Xiangyang, Hubei, China; 2 Hubei Key Laboratory of Vehicle-Infrastructure Cooperation and Traffic Control, Hubei University of Arts and Science, Xiangyang, Hubei, China; 3 School of Civil Engineering, Science and Technology College of Hubei University of Arts and Science, Xiangyang, Hubei, China; 4 CCCC First Highway Consultants Co., Ltd, Xi an, Shaanxi, China; Henan Polytechnic University, CHINA

## Abstract

To improve the durability of the chip seal and reduce the chip loss rate, a sand-containing fog seal was covered on its surface, and the response surface methodology (RSM) was adopted to optimize the mix proportion. The amount of emulsified asphalt and sand-binder ratio were selected as key factors, and experimental research was conducted with anti-skid performance, texture depth, and Vialit mass loss rate as response indicators. The results indicate that the regression model established between response values and factors exhibits good fitting degree. The increase of emulsified asphalt can significantly improve the bond strength, but it will reduce the anti-skid performance. The increase in sand-binder ratio helps to improve the pendulum friction value, but it will reduce the texture depth. For chip seal of 3–5 mm and 5–10 mm, it is recommended to use emulsified asphalt at 0.46 kg/m^2^ and 0.62 kg/m^2^, and a sand-binder ratio of 0.58 and 0.70, respectively. The optimized sand-containing fog seal exhibits good water sealing performance and reasonable open traffic time, which can significantly improve the durability and road performance of the chip seal. The research provides theoretical basis and technical support for the material design and engineering application of sand-containing fog seal.

## 1 Introduction

**Fig 1:**
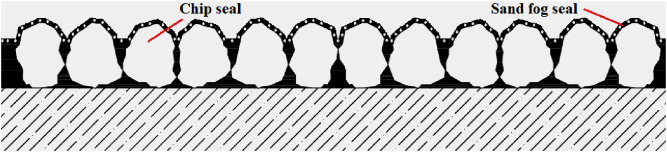
Schematic diagram of composite seal.

With the increase of road traffic volume, pavement early distresses such as cracking, raveling, and rutting in asphalt pavement occur frequently, making the importance of preventive maintenance increasingly prominent. The preventive maintenance measures for asphalt pavement mainly include techniques such as chip seal, slurry seal, micro surfacing, and ultra-thin wearing courses. As a common preventive maintenance technique, the chip seal [1] is widely used due to its advantages in waterproofing, skid resistance, easy of construction, and cost-effectiveness [2]. However, the chip seal has problems such as aggregate loss [3] and insufficient durability, especially under heavy traffic conditions, where the sealing performance deteriorates rapidly [4]. In order to improve the performance of chip seal, composite seal technology has been developed [5], as shown in Fig 1. By spraying a layer of sand-containing fog seal on the surface of chip seal, it can not only enhance the adhesion of the sealing layer, but also improve the anti-skid performance and waterproof effect [6]. Nevertheless, the material proportioning and construction process of sand-containing fog seal significantly affect its performance. If the design is not appropriate, problems such as oil bleeding, insufficient anti-skid performance, and material waste may occur [7]. Therefore, conducting research on the optimization design of sand-containing fog seal mix proportion in composite seal has important theoretical and engineering significance.

Sand-containing fog seal is recognized as an effective preventive maintenance treatment for pavement [8], which can improve the waterproof, anti-aging, adhesion and skid resistance [9]. In recent years, many scholars have conducted research on the materials and properties of fog seal. Zuo [10] developed a highly permeable fog seal material with a penetration depth ranging from 8.4 to 34.4 mm. Cai [11] developed a novel fog seal material by modifying emulsified asphalt with a composite of waterborne epoxy resin (WER), waterborne polyurethane (WPU), and waterborne acrylic resin (WAR). The pull-off strength was increased by 2–4 times. Fan [12] developed a water-based epoxy/polyurethane composite modified emulsified asphalt fog seal, which exhibited excellent resistance to long-term thermal oxidation and freeze-thaw cycles. Hu [13] determined the optimal ratio of sand-containing fog seal with a spray rate of 1.0 kg/m^2^ and a fine aggregate content of 30% for airport asphalt pavement. Previous studies have also shown that the composition and dosage of the seal have a significant impact on its performance, especially the bonding strength and skid resistance. Insufficient dosage leads to poor adhesion, while excessive dosage tends to cause bleeding and reduced skid resistance [14]. Therefore, determining the optimal ratio and dosage is crucial. In terms of optimization methods, RSM has been widely applied in mix proportion design of road materials for efficient optimization under multi-factor and multi-objective conditions. Compared to single factor experiments, RSM has many advantages, such as effectively reducing the number of experiments, exploring the interaction between factors, constructing response surfaces to achieve multi-objective optimization, and so on. For example, Saha [15] optimized the cracking performance of asphalt mixtures using RSM^.^ Hamzah [16] evaluated moisture damage properties of warm mix asphalt with this method^.^ Based on the RSM method, Ma [17] proposed an optimal ratio 21.8% water, 30% filler and 25% sand for fog seal. Li [18] integrated RSM with desirability optimization methodology (DOM) to optimize waterborne epoxy resin emulsified asphalt chip seal, identifying optimal application rates of 1.0 kg/m² for emulsified asphalt, 5.4 kg/m² for aggregates, and 6.6% for WER content. Meng [19] successfully applied RSM for anti-icing fog seal optimization, further confirming the method’s suitability for complex formulation problems involving conflicting performance objectives.

The above research has promoted the diversified development of fog seal. However, most of these studies optimize the fog seal as an independent layer, with limited attention paid to its application performance on the chip seal, which constitutes a key research gap in the application of composite seal. Therefore, this study aims to fill the gap by systematically investigating the mix proportion optimization of sand-containing fog seal applied over chip seal with different aggregate sizes. The RSM was adopted in this study to systematically investigate the effects of emulsified asphalt dosage and sand-binder ratio on the skid resistance, texture depth, and bonding performance of sand-containing fog seal covered on Chip Seal. The optimized mix proportion scheme for Sand-Containing Fog Seal applicable to different particle sizes of crushed stone chip seal proposed in this study provides scientific basis for engineering applications.

## 2 Materials

### 2.1 Emulsified asphalt

High viscosity emulsified asphalt was selected as the asphalt binder, and the technical indicators are shown in Table 1.

**Table 1:** Technical indicators of high-viscosity emulsified asphalt.

Technical indicator		Unit	Test result	Requirement	Test method
Residue on 1.18 mm sieve		%	0.071	≤0.1	T0652
Englar viscosity E_25_		–	7.32	1 ∼ 10	T0622
Evaporated residue content		%	62.5	≥50	T0651
Evaporated residue	Penetration (25℃)	0.1mm	59.3	40 ∼ 120	T0604
	Ductility (5℃)	cm	31.8	≥20	T0605
	Softening point	℃	61.8	≥50	T0606
Storage stability	1d	%	0.62	≤1	T0655
	5d	%	2.1	≤5	T0655

### 2.2 Aggregate

The aggregate type used for the chip seal in this study is basalt, including two specifications of 3–5 mm and 5–10 mm. Its technical indicators are shown in Table 2.

**Table 2:** Technical indicators of aggregate.

Technical Indicator	Unit	3 ∼ 5 mm	5 ∼ 9.5 mm
Los Angeles abrasion value	%	11.5	
Crushing value	%	9.8	
Water absorption rate	%	0.75	
Bulk density	$\text{g}/{\text{cm}}^{\text{3}}$	2.55	2.75
Needle flake content	%	2.45	3.08

### 2.3 Sand

The Carborundum is selected as the sand in the fog seal. It has the characteristics of high hardness, good impact resistance and wear resistance, and is commonly used as anti-skid fine aggregate for roads. The basic technical indicators are shown in Table 3.

**Table 3:** Basic technical indicators of carborundum.

Mesh Size	SiC Content (%)	Free Carbon Content (%)	Moisture Content (%)	Apparent Relative Density (g/cm^3^)	Mohs Hardness
30 ~ 100	>98	0.27	0.03	3.52	9.1

## 3 Experimental

### 3.1 Response surface experimental design

In the central composite design (CCD) mode of RSM, the emulsified asphalt spraying amount (0.3–0.7 kg/m^2^ for 3−5 mm chip seal and 0.5–0.9 kg/m^2^ for 5−10 mm chip seal) and sand-binder ratio (0.2–0.8) were selected as two factors, denoted as X_1_ and X_2_, respectively. Three evaluation indicators, namely the British pendulum Number (BPN), Mean Texture Depth (MTD), and mass loss rate, were taken as response values. Thirteen sets of experiments were designed using Design Expert software. The factor level and value of CCD are shown in Table 4.

**Table 4:** Factor levels and values of CCD.

Factor		−1.414	−1	0	+1	+1.414
X_1_/ Emulsified Asphalt Dosage (kg/m^2^)	3 ∼ 5 mm	0.30	0.36	0.50	0.64	0.70
	5 ∼ 10 mm	0.50	0.56	0.70	0.84	0.90
X_2_ / Sand-Binder Ratio		0.20	0.29	0.50	0.71	0.80

**Fig 2:**
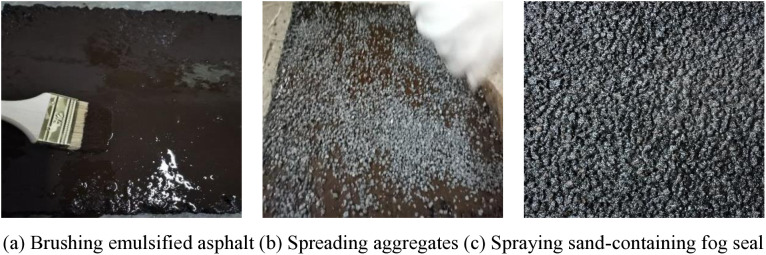
Preparation process of sand-containing fog seal specimens.

The test pieces were formed according to the above scheme. Firstly, the asphalt rutting plate specimens were prepared as the carrier for applying the seal. Then, 3 ~ 5 mm and 5 ~ 10 mm chip seal layers are paved on the rutting plate respectively. For the chip seal of 3–5 mm and 5–10 mm, the amount of basalt aggregate used is 6.49 kg/m^2^ and 11.41 kg/m^2^, respectively. The amount of emulsified asphalt used is 1.53 kg/m^2^ and 1.79 kg/m^2^, respectively. Finally, a sand-containing fog seal is spread on top of the chip seal. After preparation, the specimens were cured at room temperature for 24h, and the test process is shown in Fig 2.

### 3.2 Pendulum value test

A British Pendulum friction tester was used to determine the BPN of different specimens. Three parallel tests were conducted, and the average value was taken as the test result. The ambient temperature must be strictly controlled at 20℃ when the pendulum friction tester was used to measure the BPN in this experiment. The pendulum values BPN_T_ measured at different ambient temperatures were converted into the pendulum values BPN_20_ at the standard temperature of 20 ℃ according to the following formula (1).


$$\begin{array}{c} {BPN}_{\text{2}\text{0}}={BPN}_{T}+\Delta BPN \end{array}$$ (1)

Where: BPN_20_ – pendulum value at standard temperature 20°C; BPN_T_ – pendulum value at ambient temperature T °C; ΔBPN – temperature correction value, adopted according to Table 5.

**Table 5:** Temperature correction values for pendulum value.

Temperature (°C)	0	5	10	15	20	25	30	35	40
Temperature Correction Value ΔBPN	−6	−4	−3	−1	0	+2	+3	+5	+7

### 3.3 Texture depth test

According to the Field Test Methods of Highway Subgrade and Pavement (JTG 3450−2019) [20] the texture depth was measured using the sand patch test. Two parallel tests were conducted, and the average value was taken as the test result. The MTD is calculated by the following formula (2).


$$\begin{array}{c} \text{MTD}=\frac{\text{1000V}}{\pi {\text{D}}^{\text{2}}/\text{4}} \end{array}$$ (2)

Where: MTD – the mean texture depth, mm; V- volume of sand used in sand patch test, generally 25 cm^2^; D – average diameter of sand after being flattened into a circle, mm.

### 3.4 Vialit test

The Vialit test was used to evaluate the enhancement of sand-containing fog seal on the bonding strength of chip seal, and the steps of Vialit test are as follows:

(1) The mass of the rutting plate test mold was weighed and recorded as m_0_;
(2) After the composite seal test piece was formed, the total mass of the test piece and the mold was weighed and recorded as m_1_;
(3) At room temperature, the mold with the test piece was turned over and placed on the test bench, and a 500g steel ball fell from a height of 50 cm to hit the center of the test mold. The falling of the aggregate was observed, and the remaining mass of the test piece was weighed and recorded as m_2_;
(4) The mass loss rate AR was calculated according to the following formula (3).

$$\begin{array}{c} \text{AR}=\frac{{\text{m}}_{\text{1}}-{\text{m}}_{\text{2}}}{{\text{m}}_{\text{1}}-{\text{m}}_{\text{0}}}\times \text{100}\% \end{array}$$ (3)

## 4 Results and discussion

### 4.1 Experimental results

The test results are shown in Table 6.

**Table 6:** Response surface experimental results.

Number	Factors		3 ∼ 5 mm			5 ∼ 10 mm		
	$X_{1}$	$X_{2}$	${BPN}_{20}$	MTD/mm	AR /%	${BPN}_{20}$	MTD/mm	AR /%
1	−1	−1	97.0	1.73	8.6	105.2	2.69	7.4
2	1	−1	95.8	1.43	2.9	103.8	2.51	1.6
3	−1	1	103.2	1.69	8.9	112.8	2.58	8.0
4	1	1	100.0	1.38	3.2	105.2	2.34	1.9
5	−1.414	0	102.5	1.83	12.8	108.5	2.81	11.5
6	1.414	0	96.8	1.23	0.7	102.6	2.31	0.2
7	0	−1.414	94.6	1.63	5.9	101.4	2.78	5.2
8	0	1.414	104.0	1.28	6.4	109.8	2.41	5.8
9	0	0	101.3	1.56	6.2	104.8	2.64	5.5
10	0	0	101.6	1.58	6.1	104.5	2.62	5.4
11	0	0	101.1	1.54	6.3	105.4	2.66	5.6
12	0	0	101.4	1.56	6.2	104.2	2.62	5.5
13	0	0	100.8	1.54	6.4	105.8	2.64	5.3

According to the experimental results in Table 6, the second-order polynomial relationship model between the factors X_1_, X_2_ and the response values BPN_20_, MTD and AR was obtained by regression analysis with the design expert software.

(1) 3–5 mm composite seal:


$$\begin{array}{c} {\text{BPN}}_{\text{20}}=\text{101}.\text{24}-\text{1}.\text{56}{\text{X}}_{\text{1}}+\text{2}.\text{96}{\text{X}}_{\text{2}}-\text{0}.\text{5}{\text{X}}_{\text{1}}{\text{X}}_{\text{2}}-\text{0}.\text{91}{{\text{X}}_{\text{1}}}^{\text{2}}-\text{1}.\text{09}{{\text{X}}_{\text{2}}}^{\text{2}} \end{array}$$

$$\begin{array}{c} \text{MTD}=\text{1}.\text{56}-\text{0}.\text{18}{\text{X}}_{\text{1}}-\text{0}.\text{07}{\text{X}}_{\text{2}}-\text{0}.\text{002}{\text{X}}_{\text{1}}{\text{X}}_{\text{2}}+\text{0}.\text{003}{{\text{X}}_{\text{1}}}^{\text{2}}-\text{0}.\text{03}{{\text{X}}_{\text{2}}}^{\text{2}} \end{array}$$

$$\begin{array}{c} \text{AR}=\text{6}.\text{24}-\text{3}.\text{56}{\text{X}}_{\text{1}}+\text{0}.\text{16}{\text{X}}_{\text{2}}+\text{0}.\text{00}{\text{X}}_{\text{1}}{\text{X}}_{\text{2}}+\text{0}.\text{12}{{\text{X}}_{\text{1}}}^{\text{2}}-\text{0}.\text{18}{{\text{X}}_{\text{2}}}^{\text{2}} \end{array}$$

(2) 5–10 mm composite seal:


$$\begin{array}{c} {\text{BPN}}_{\text{20}}=\text{104}.\text{94}-\text{2}.\text{17}{\text{X}}_{\text{1}}+\text{2}.\text{61}{\text{X}}_{\text{2}}-\text{1}.\text{55}{\text{X}}_{\text{1}}{\text{X}}_{\text{2}}+\text{0}.\text{60}{{\text{X}}_{\text{1}}}^{\text{2}}+\text{0}.\text{62}{{\text{X}}_{\text{2}}}^{\text{2}} \end{array}$$

$$\begin{array}{c} \text{MTD}=\text{2}.\text{64}-\text{0}.\text{14}{\text{X}}_{\text{1}}-\text{0}.\text{10}{\text{X}}_{\text{2}}-\text{0}.\text{01}{\text{X}}_{\text{1}}{\text{X}}_{\text{2}}-\text{0}.\text{05}{{\text{X}}_{\text{1}}}^{\text{2}}-\text{0}.\text{03}{{\text{X}}_{\text{2}}}^{\text{2}} \end{array}$$

$$\begin{array}{c} \text{AR}=\text{18}.\text{60}-\text{19}.\text{15}{\text{X}}_{\text{1}}\pm \text{8}.\text{21}{\text{X}}_{\text{2}}-\text{2}.\text{55}{\text{X}}_{\text{1}}{\text{X}}_{\text{2}}-\text{3}.\text{19}{{\text{X}}_{\text{1}}}^{\text{2}}-\text{3}.\text{18}{{\text{X}}_{\text{2}}}^{\text{2}} \end{array}$$

In order to perform error statistical analysis on the regression equation, some parameters of the fitting model are listed in Table 7, including root mean square error (RMSE), mean absolute error (MAE), mean absolute percentage error (MAPE), R², coefficient of variation (C.V.), F value, and P value.

**Table 7:** Fitting analysis results of each regression equation.

Response target		Fitting parameters					C.V./%	$R^{2}$
Particle Size	Index	F Value	P Value	*RMSE*	*MAE*	*MAPE/%*		
3 ∼ 5 mm	${BPN}_{\text{20}}$	40.69	<0.0001	0.52	0.43	0.43	0.71	0.967
	MTD	11.95	0.0026	0.05	0.04	2.78	4.74	0.895
	AR	30.21	0.0001	0.60	0.45	14.03	13.27	0.956
5 ∼ 10 mm	${BPN}_{\text{20}}$	26.75	0.0002	0.65	0.56	0.54	0.84	0.950
	MTD	15.74	0.0011	0.04	0.03	1.27	2.23	0.918
	AR	34.80	<0.0001	0.55	0.38	14.51	14.15	0.961

From Table 7, it can be seen that all P-values are less than 0.05, R^2^ is greater than 0.9, RMSE and MAE are less than 1.0, indicating good reliability of the experimental results and high degree of model fitting. Further analysis shows that the F-value and MAPE of AR are the highest and the P-value is the lowest among all response indicators, indicating the strongest correlation between AR and particle size.

**Fig 3:**
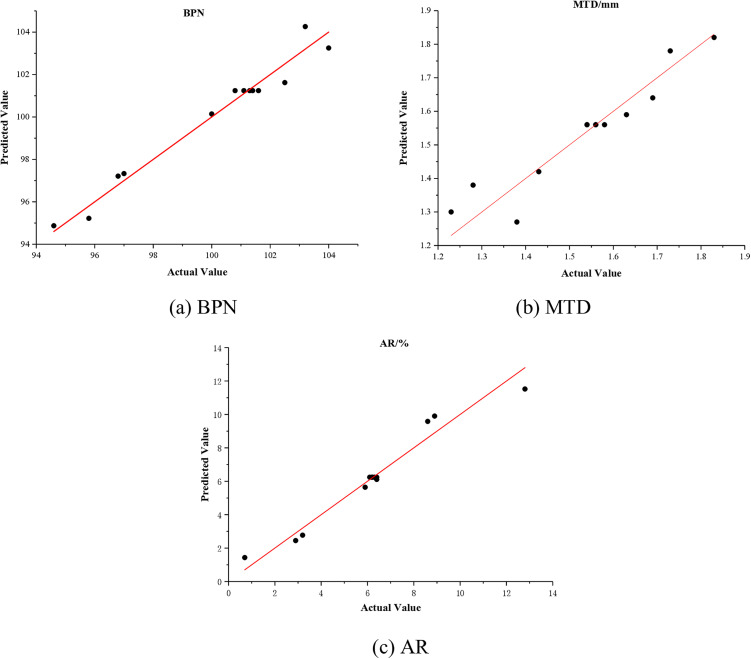
Comparison of model predicted and experimental values for 3-5 mm particle size composite seal.

**Fig 4:**
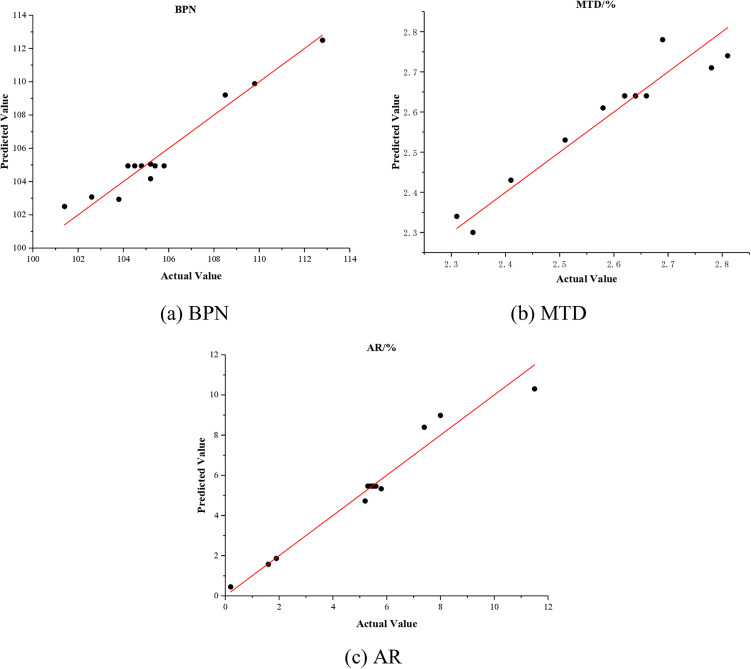
Comparison of model predicted and experimental values for 5-10 mm particle size composite seal.

Through multiple regression analysis, the predicted values of BPN_20_, MTD, and AR were compared with the experimental values, as shown in Figs 3 and 4.

From Figs 3 and 4, it can be seen that the predicted values of BPN_20_, MTD, and AR models are close to the experimental values, indicating high reliability and accuracy of the models.

### 4.2 Interaction effect

**Fig 5:**
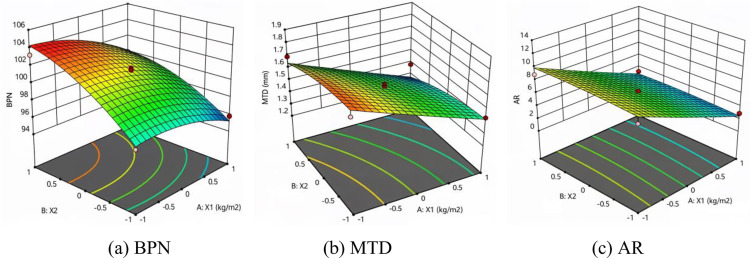
Three-dimensional response surface of 3–5 mm composite seal.

**Fig 6:**
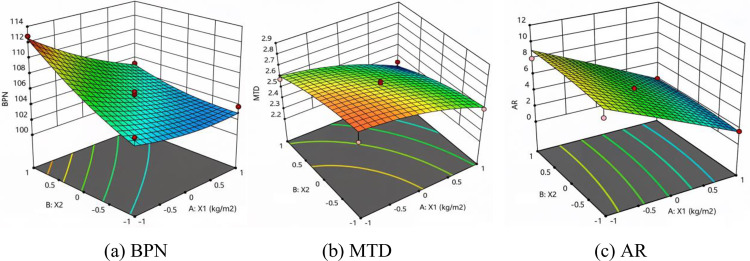
Three-dimensional response surface of 5–10 mm composite seal.

The three-dimensional response surfaces of BPN_20_, MTD, and AR of the composite seal layer under the interaction of emulsified asphalt dosage and sand-binder ratio are shown in Figs 5 and 6.

From Figs 5 and 6, it can be seen that the amount of emulsified asphalt has a significant impact on the bonding performance of the composite seal layer. As the amount increases, the quality loss rate AR gradually decreases, indicating that emulsified asphalt enhances the overall bonding strength of the aggregate. Mechanistically, more emulsified asphalt can form a sufficient asphalt film, fully wrapping the aggregate of the chip seal and SiC anti-skid fine sand, which effectively limits the detachment of particles under vehicle load, thereby reducing the quality loss rate of the aggregate. In addition, as the amount of emulsified asphalt increases, the BPN_20_ and MTD of the composite seal significantly decrease, indicating that excessive application of emulsified asphalt can reduce the anti-skid performance of the composite seal. The excess asphalt will cover the tiny protrusions of SiC sand and the microtexture of the aggregate. The lack of microtexture and filling of gaps can reduce the friction between the tire and the road surface, resulting in the decrease of BPN_20_ and MTD. Moreover, excessive emulsified asphalt can also cause the composite seal to exhibit phenomena such as wheel sticking and oil bleeding during high temperatures in summer. Therefore, from the perspective of anti-skid performance, the amount of emulsified asphalt should not be too much.

The sand-binder ratio has a significant impact on the BPN_20_ and MTD of the composite seal. As the sand-binder ratio increases, the BPN_20_ significantly increases. This is mainly because SiC sand has high Mohs hardness and sharp micro convex structure. Increasing the sand-binder ratio will increase the number of anti-skid particles on the road surface, thereby enhancing the friction between the tire and the seal, directly improving the BPN_20_ value. When the sand-binder ratio of the 3–5 mm composite seal layer reaches around 0.50 and the sand-binder ratio of the 5–10 mm composite seal layer reaches around 0.60, the BPN_20_ growth slows down, indicating that the improvement in the anti-skid performance of the composite seal layer is not significant thereafter. The MTD gradually decreases with the increase of the sand-binder ratio, mainly because the sand will gradually fill the small gaps between the crushed stones, and the larger the sand-binder ratio, the more obvious the filling effect, thereby reducing the MTD of the composite sealing layer.

### 4.3 Determination of optimal ratio

Design Expert software was used for optimization. Within the range of experimental factors, the response values of BPN_20_ and MTD were set to the maximum and the mass loss rate AR was set to the minimum, and the optimal ratio was obtained by solving the model.

**Fig 7:**
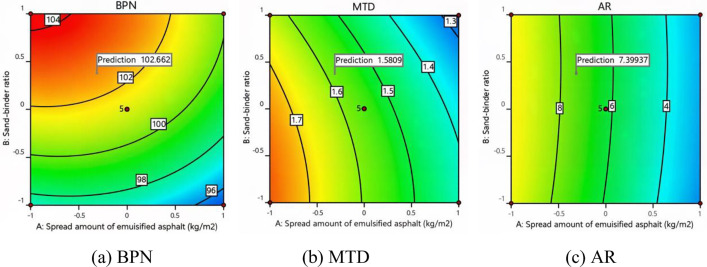
Optimal mix ratio prediction for 3–5 mm composite seal.

For the 3–5 mm composite seal, the optimized emulsified asphalt spraying amount and sand-binder ratio are 0.46 kg/m^2^ and 0.58, respectively. The corresponding BPN prediction value is 102.662, MTD is 1.581, and AR is 7.399%, as shown in Fig 7.

**Fig 8:**
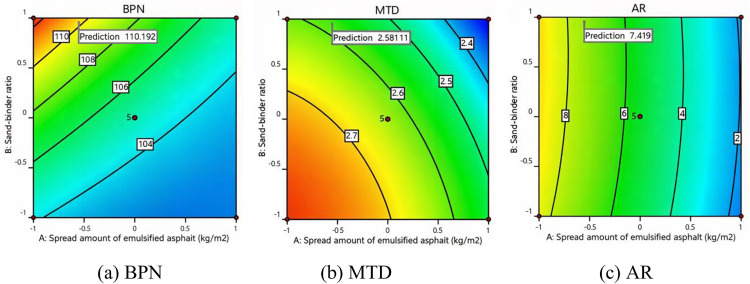
Optimal mix ratio prediction for 5–10 mm composite seal.

For the 5–10 mm composite seal, the optimized emulsified asphalt spraying amount and sand-binder ratio are 0.62 kg/m^2^ and 0.70, respectively. The corresponding BPN prediction value is 110.192, MTD is 2.581, and AR is 7.419%, as shown in Fig 8.

## 5 Performance evaluation of composite seal

### 5.1 Water sealing performance

**Fig 9:**
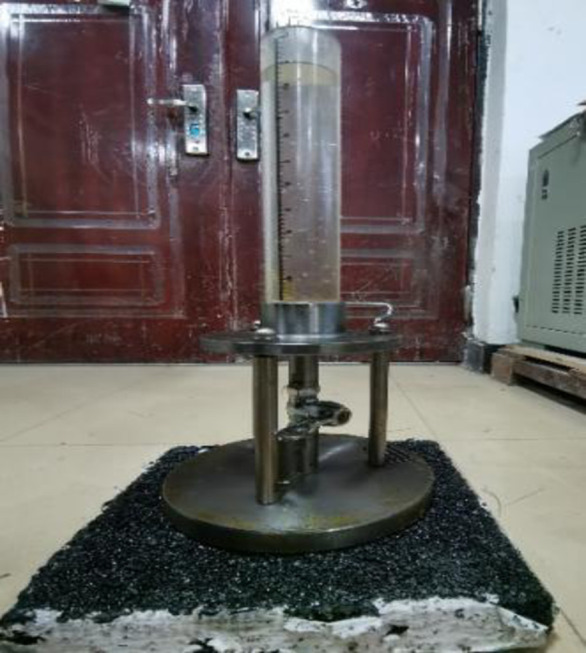
Pavement permeability test.


The pavement water permeability meter was used to measure the water permeability coefficient of the rutting plates with chip seal and those with composite seal (as shown in Fig 9), respectively. The permeability coefficient of each specimen was calculated using the following formula to evaluate the sealing performance (4).

$$\begin{array}{c} C_{w}=\frac{V_{\text{2}}-V_{\text{1}}}{t_{\text{2}}-t_{\text{1}}}\times \text{60} \end{array}$$ (4)

Where:$C_{w}$- permeability coefficient,ml/min; $V_{\text{1}}$- water volume in the measuring cylinder at the beginning,ml; $V_{\text{2}}$- water volume in the measuring cylinder at the end,ml; $t_{\text{1}}$- start time,s; $t_{\text{2}}$- end time,s.

The permeability test results are shown in Table 8.

**Table 8:** Permeability test results of different seal types.

Particle Size/mm	Seal type	Permeability coefficient / (ml/min)	Permeability status
3 ∼ 5 mm	Chip Seal	2.5	Slight Permeability
	Composite Seal	0.2	No Permeability
5 ∼ 10 mm	Chip Seal	16.2	Permeable
	Composite Seal	0.3	Almost No Permeability

As shown in Table 8, the permeability coefficient of the 5–10 mm chip seal is much higher than that of the 3–5 mm chip seal. This is because the larger the particle size, the rougher the surface of the seal, and the larger the gaps, resulting in poorer water sealing ability. In addition, the permeability coefficient of the composite seal is much lower than that of the chip seal with the same particle size, indicating better water sealing performance of the composite seal. This is mainly because the sand-containing fog seal can effectively fill the gaps in the chip seal, thereby improving the sealing effect.

### 5.2 Time of traffic opening

Determining the traffic opening time is a crucial step after applying the composite seal on asphalt pavement. If traffic is opened too early, due to incomplete demulsification of emulsified asphalt, the bonding performance between asphalt and aggregate is insufficient, and the composite seal is prone to suffer from early diseases such as aggregate peeling and pushing under the action of vehicle loads. If traffic is opened too late, it will be difficult to achieve the goal of rapid traffic opening and may lead to traffic congestion. The curing of the composite seal generally includes two processes: surface drying and complete drying. Surface drying time is the initial setting time of the material, which is far from the formation of material strength. Complete drying time is the time from application to the basic formation of strength. The specific test procedures are as follows:

(1) According to the optimized mix proportion, composite seal specimens with different particle sizes are formed;
(2) To simulate the curing time of composite seal at different temperatures, the specimens were placed in ovens at 25 ℃, 40 ℃, and 60 ℃;
(3) At regular intervals, the specimens were taken out to observe their surface condition and determine the surface drying time and complete drying time of different specimens.

The experimental results are shown in Table 9.

**Table 9:** Surface drying and complete drying times at different temperatures.

Seal Type	Index	Temperature/℃		
		25	40	60
3-5 mm Composite Seal	Surface Drying Time	2.5h	1.0h	40min
	Complete Drying Time	9h	6.5h	3.5h
5-10 mm Composite Seal	Surface Drying Time	3.0h	1.0h	30min
	Complete Drying Time	8.5h	6h	3.5h

From Table 9, it can be seen that the surface drying time and complete drying time of composite seals with the same particle size gradually decrease with increasing temperature. This is mainly because the increase in temperature accelerates the evaporation of water in the emulsified asphalt, speeding up the demulsification process and thereby reducing the surface drying time and the complete drying time. In addition, there is not much difference in surface drying time and complete drying time between composite seals with particle sizes of 3–5 mm and 5–10 mm. This is mainly because the amount of emulsified asphalt used in composite sealing layers with different particle sizes is different, and the thickness of the film formed by emulsified asphalt on the surface is not significantly different. Therefore, the particle size of the aggregate has little effect on the surface drying time and complete drying time.

## 6 Conclusions

Based on response surface methodology, the effects of emulsified asphalt dosage and sand-binder ratio on the anti-skid performance, texture depth, and Vialit mass loss rate of sand-containing fog seal were studied. The optimal ratio was optimized, and the sealing performance and open traffic time were studied. The main conclusions are as follows:

(1) With the increase of emulsified asphalt dosage, the bonding strength of the seal can be significantly improved, but excessive dosage can lead to a decrease in anti-skid performance; An appropriate sand-binder ratio can help improve the BPN, but an excessive ratio will reduce the MTD.
(2) For composite seal of 3–5 mm and 5–10 mm, the recommended emulsified asphalt application rates are 0.46 kg/m² and 0.62 kg/m², respectively, with sand-binder ratio of 0.58 and 0.70, respectively.
(3) The optimized composite seal has good water sealing performance and reasonable open traffic time, which can significantly improve the durability and road performance of the chip seal.

## Supporting information

S1 File Raw data set. (DOCX)
