# Supplementary material for: Optimization design of sand-containing fog seal overlay on chip seal based on response surface methodology
Source: PLoS One. 2026 Aug 12;21(8):e0355403. doi: 10.1371/journal.pone.0355403 (PMC13465809; doi:10.1371/journal.pone.0355403)
Supplement: Supplementary file 1 [file pone.0355403.s001.docx]

**The data of 3～5mm chip seal at 20℃ in Table 6**

| Number | BPN1 | BPN2 | BPN3 | Average of BPN | MTD1/mm | MTD2/mm | Average of MTD/mm | AR/% |
| --- | --- | --- | --- | --- | --- | --- | --- | --- |
| 1 | 96.3 | 97.5 | 97.2 | 97.0 | 1.71 | 1.75 | 1.73 | 8.6 |
| 2 | 95.1 | 96.4 | 95.9 | 95.8 | 1.38 | 1.48 | 1.43 | 2.9 |
| 3 | 102.5 | 103.7 | 103.4 | 103.2 | 1.67 | 1.71 | 1.69 | 8.9 |
| 4 | 99.3 | 100.6 | 100.1 | 100.0 | 1.39 | 1.37 | 1.38 | 3.2 |
| 5 | 101.8 | 103 | 102.7 | 102.5 | 1.85 | 1.81 | 1.83 | 12.8 |
| 6 | 96.4 | 96.7 | 97.3 | 96.8 | 1.19 | 1.27 | 1.23 | 0.7 |
| 7 | 93.8 | 95.4 | 94.6 | 94.6 | 1.61 | 1.65 | 1.63 | 5.9 |
| 8 | 103.2 | 104.7 | 104.1 | 104.0 | 1.26 | 1.30 | 1.28 | 6.4 |
| 9 | 100.5 | 101.8 | 101.6 | 101.3 | 1.58 | 1.54 | 1.56 | 6.2 |
| 10 | 101.9 | 100.6 | 102.3 | 101.6 | 1.54 | 1.62 | 1.58 | 6.1 |
| 11 | 100.3 | 101.5 | 101.5 | 101.1 | 1.55 | 1.53 | 1.54 | 6.3 |
| 12 | 101.5 | 100.9 | 101.8 | 101.4 | 1.57 | 1.55 | 1.56 | 6.2 |
| 13 | 100.4 | 100.8 | 101.2 | 100.8 | 1.50 | 1.58 | 1.54 | 6.4 |

**The data of 5～10mm chip seal at 20℃ in Table 6**

| Number | BPN1 | BPN2 | BPN3 | Average of BPN | MTD1/mm | MTD2/mm | Average of MTD/mm | AR/% |
| --- | --- | --- | --- | --- | --- | --- | --- | --- |
| 1 | 104.2 | 105.8 | 105.6 | 105.2 | 2.65 | 2.73 | 2.69 | 7.4 |
| 2 | 103.7 | 103.5 | 104.2 | 103.8 | 2.47 | 2.55 | 2.51 | 1.6 |
| 3 | 112.1 | 113.1 | 113.2 | 112.8 | 2.62 | 2.54 | 2.58 | 8.0 |
| 4 | 104.7 | 105.3 | 105.6 | 105.2 | 2.36 | 2.32 | 2.34 | 1.9 |
| 5 | 107.9 | 109.1 | 108.5 | 108.5 | 2.79 | 2.83 | 2.81 | 11.5 |
| 6 | 101.3 | 103.2 | 103.3 | 102.6 | 2.33 | 2.29 | 2.31 | 0.2 |
| 7 | 100.5 | 101.8 | 101.9 | 101.4 | 2.74 | 2.82 | 2.78 | 5.2 |
| 8 | 108.9 | 110.4 | 110.1 | 109.8 | 2.43 | 2.39 | 2.41 | 5.8 |
| 9 | 104.3 | 104.7 | 105.4 | 104.8 | 2.62 | 2.66 | 2.64 | 5.5 |
| 10 | 104.8 | 103.6 | 105.1 | 104.5 | 2.65 | 2.59 | 2.62 | 5.4 |
| 11 | 105.1 | 105.8 | 105.3 | 105.4 | 2.58 | 2.74 | 2.66 | 5.6 |
| 12 | 104.2 | 103.6 | 104.8 | 104.2 | 2.64 | 2.60 | 2.62 | 5.5 |
| 13 | 104.4 | 106.3 | 106.7 | 105.8 | 2.61 | 2.67 | 2.64 | 5.3 |

**The data of BPN in Figure 3 (a)**

| Number of experiments | Actual Value | Predicted Value |
| --- | --- | --- |
| 1 | 100.0 | 100.14 |
| 2 | 101.3 | 101.24 |
| 3 | 97.0 | 97.33 |
| 4 | 103.2 | 104.26 |
| 5 | 101.6 | 101.24 |
| 6 | 101.1 | 101.24 |
| 7 | 102.5 | 101.62 |
| 8 | 101.4 | 101.24 |
| 9 | 104.0 | 103.25 |
| 10 | 94.6 | 94.87 |
| 11 | 96.8 | 97.21 |
| 12 | 95.8 | 95.22 |
| 12 | 100.8 | 101.24 |

**The data of MTD in Figure 3 (b)**

| Number of experiments | Actual Value | Predicted Value |
| --- | --- | --- |
| 1 | 1.38 | 1.27 |
| 2 | 1.56 | 1.56 |
| 3 | 1.73 | 1.78 |
| 4 | 1.69 | 1.64 |
| 5 | 1.58 | 1.56 |
| 6 | 1.54 | 1.56 |
| 7 | 1.83 | 1.82 |
| 8 | 1.56 | 1.56 |
| 9 | 1.28 | 1.38 |
| 10 | 1.63 | 1.59 |
| 11 | 1.23 | 1.30 |
| 12 | 1.43 | 1.42 |
| 12 | 1.54 | 1.56 |

**The data of AR in Figure 3 (c)**

| Number of experiments | Actual Value | Predicted Value |
| --- | --- | --- |
| 1 | 3.2 | 2.77 |
| 2 | 6.2 | 6.24 |
| 3 | 8.6 | 9.58 |
| 4 | 8.9 | 9.90 |
| 5 | 6.1 | 6.24 |
| 6 | 6.3 | 6.24 |
| 7 | 12.8 | 11.52 |
| 8 | 6.2 | 6.24 |
| 9 | 6.4 | 6.11 |
| 10 | 5.9 | 5.64 |
| 11 | 0.7 | 1.43 |
| 12 | 2.9 | 2.45 |
| 12 | 6.4 | 6.24 |

**The data of BPN in Figure 4 (a)**

| Number of experiments | Actual Value | Predicted Value |
| --- | --- | --- |
| 1 | 104.8 | 104.94 |
| 2 | 103.8 | 102.93 |
| 3 | 105.2 | 105.05 |
| 4 | 104.5 | 104.94 |
| 5 | 101.4 | 102.50 |
| 6 | 102.6 | 103.07 |
| 7 | 105.2 | 104.17 |
| 8 | 109.8 | 109.88 |
| 9 | 105.4 | 104.94 |
| 10 | 108.5 | 109.20 |
| 11 | 104.2 | 104.94 |
| 12 | 105.8 | 104.94 |
| 12 | 112.8 | 112.49 |

**The data of MTD in Figure 4 (b)**

| Number of experiments | Actual Value | Predicted Value |
| --- | --- | --- |
| 1 | 2.64 | 2.64 |
| 2 | 2.51 | 2.53 |
| 3 | 2.34 | 2.30 |
| 4 | 2.62 | 2.64 |
| 5 | 2.78 | 2.71 |
| 6 | 2.31 | 2.34 |
| 7 | 2.69 | 2.78 |
| 8 | 2.41 | 2.43 |
| 9 | 2.66 | 2.64 |
| 10 | 2.81 | 2.74 |
| 11 | 2.62 | 2.64 |
| 12 | 2.64 | 2.64 |
| 12 | 2.58 | 2.61 |

**The data of AR in Figure 4 (c)**

| Number of experiments | Actual Value | Predicted Value |
| --- | --- | --- |
| 1 | 5.5 | 5.46 |
| 2 | 1.6 | 1.57 |
| 3 | 1.9 | 1.86 |
| 4 | 5.4 | 5.46 |
| 5 | 5.2 | 4.72 |
| 6 | 0.2 | 0.45 |
| 7 | 7.4 | 8.39 |
| 8 | 5.8 | 5.33 |
| 9 | 5.6 | 5.46 |
| 10 | 11.5 | 10.3 |
| 11 | 5.5 | 5.46 |
| 12 | 5.3 | 5.46 |
| 12 | 8.0 | 8.98 |
